# An Active Isodicentric X Chromosome in a Case of Refractory Anaemia with Ring Sideroblasts Associated with Marked Thrombocytosis

**DOI:** 10.1155/2014/205318

**Published:** 2014-01-30

**Authors:** Rosario M. Morales Camacho, Javier Sanchez, Irene Marcos Luque, Ricardo Bernal, Jose F Falantes, Jose A Pérez-Simón

**Affiliations:** ^1^Department of Haematology, Institute of Biomedicine of Seville (IBIS), University Hospital Virgen del Rocío, CSIC, University of Seville, 41013 Seville, Spain; ^2^Department of Genetics, Reproduction and Fetal Medicine, Institute of Biomedicine of Seville (IBIS), University Hospital Virgen del Rocío, CSIC, University of Seville, 41013 Seville, Spain; ^3^Centre of Biomedical Network Research on Rare Diseases (CIBERER), 41013 Seville, Spain

## Abstract

Refractory anaemia with ring sideroblasts and marked thrombocytosis (RARS-T) is a provisional entity in the World Health Organization (WHO) classification. It displays features characteristic of both myelodysplastic syndrome and myeloproliferative neoplasia plus ring sideroblasts ≥15% and marked thrombocytosis. Most patients with RARS-T show a normal karyotype. We report a 76-year-old woman diagnosed with RARS-T (76% of ring sideroblasts) with *JAK2* (V617F) mutation and a load of 30–40%. Classical and molecular cytogenetic (FISH) studies of a bone marrow sample revealed the presence of isodicentric X chromosome [(idic(X)(q13)]. Moreover, HUMARA assay showed the idic(X)(q13) as the active X chromosome. This finding was correlated with the cytochemical finding of ring sideroblasts. To our knowledge, this is the first reported case of an active isodicentric X in a woman with RARS-T.

## 1. Introduction

Refractory anaemia with ring sideroblasts and marked thrombocytosis (RARS-T) is a rare entity which displays features characteristic of both myelodysplastic syndrome and myeloproliferative neoplasia plus ring sideroblasts ≥15% and marked thrombocytosis. Most patients with RARS-T show a normal karyotype in bone marrow sample [1]. Seven years after its incorporation into the 2001 World Health Organization (WHO) classification [2], RARS-T still remains a provisional entity under the category of myelodysplastic/myeloproliferative neoplasm, unclassifiable [3].

An isodicentric X chromosome with breakpoints in Xq13 (idic(X)(q13)) is a rare cytogenetic abnormality with an extra dose of Xpter-q13 and loss of the Xq13-qter region. Currently, controversy exists regarding whether idic(X)(q13) is active or inactive and whether or not there is any correlation with the cytochemical finding of ring sideroblasts [4].

## 2. Case Report

**Figure 1:**
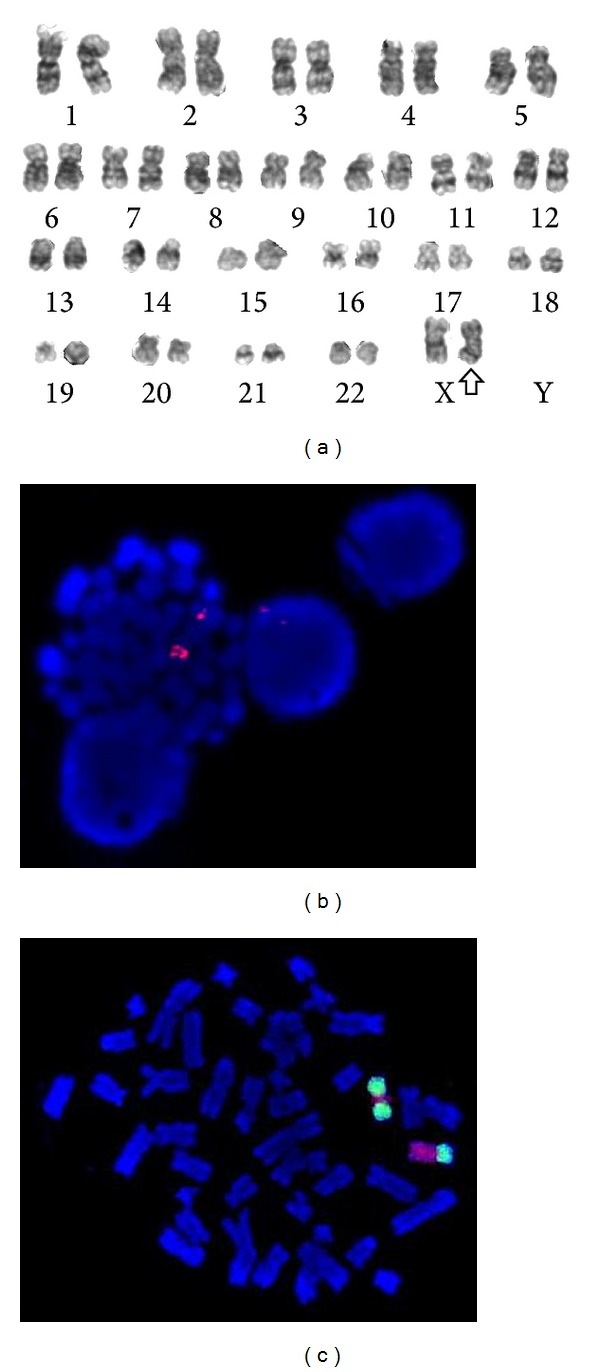
(a) G-banded karyotype of the bone marrow cells showed a 46,X,idic(X)(q13)[4]/46,XX[16]. The arrow indicates the isodicentric X chromosome. (b) FISH analysis in metaphase cell with CEP X probe showed two centromeres on the idic(X), red signals. (c) Hybridisation on metaphase cell with WCP Xp (green signal) and WCP Xq probes (red signal) showed a normal X chromosome and an idic(X) with a red signal (Xq) between two green signals (Xp).

We report a 76-year-old female presenting with RARS-T and idic(X)(q13) in bone marrow cells. She presented with anaemia (mean corpuscular volume, 103 fl; haemoglobin, 9.2 g/dL), leukocytes of 11.7 × 10^9^/L, and a thrombocytosis of 709 × 10^9^/L. The patient was diagnosed with RARS-T (76% of ring sideroblasts) with *JAK2* (V617F) mutation and a load of 30%–40%. A cytogenetic analysis of the bone marrow revealed two clones, one with an isodicentric X and another with a normal chromosomal complement: 46,X,idic(X)(q13)[4]/46,XX[16] (Figure 1(a)). HUMARA assay showed the idic(X)(q13) as the active X chromosome. *In situ* fluorescent hybridisation with a DXZ1 centromere probe (Vysis Downers Grove, IL, USA) (Figure 1(b)) and a whole chromosome painting (WCP) X chromosome probe (Vysis Downers Grove, IL, USA) (Figure 1(c)) showed two centromeres and the duplication of Xpter-q13. Cytogenetic analysis performed in a 72-hour lymphocyte culture obtained from whole blood stimulated with PHA was normal.

A CGH array performed with human CGH 720K Whole Genome Tiling v3.0 array HG 18 (Nimblegen, Roche) did not detect any abnormality in the number of copies of the X chromosome, which may be due to the fact that idic(X)(q13) was only present in a small subclone (4/20 metaphases).

The X chromosome inactivation (XCI) tests consisted of examining the methylation state of the human androgen receptor gene (*HUMARA*, Xq12), as described by Allen et al. [5]. This analysis allows the identification of the active X chromosome by analysing the methylation state of a highly polymorphic and typically heterozygotic region (CAG)n at the 5′ end of this gene, in the excess region of the isodicentric chromosome. Bone marrow samples were separated using an automated DNA sequencer (ABI3730, Life Technologies, Carlsbad, CA) and analysed by GeneMapper software (Life Technologies) for peak positioning and calculating the peak areas. These data were used to calculate the XCI ratio.

**Figure 2:**
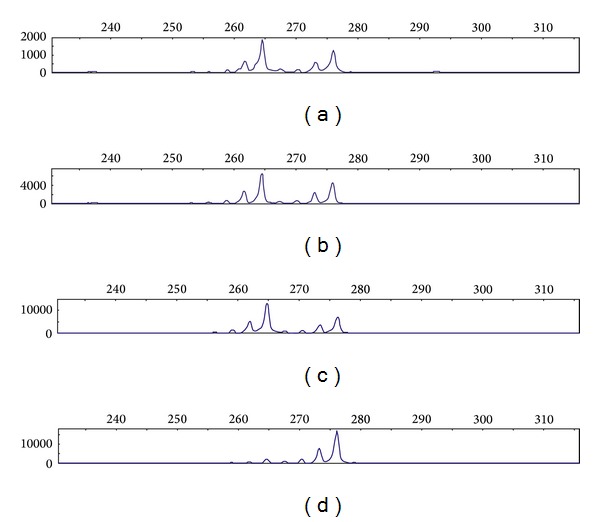
HUMARA analysis. The panels show size-separation of PCR products by capillary electrophoresis. The *x*-axes show the sizes in base pairs and the *y*-axes the fluorescence activity. Each peak corresponds to one allele of the amplified microsatellite. Controls (a) and (b): random methylation pattern: no difference observed between the undigested sample (a) and digested sample (b). Patients (c) and (d). (c) Genomic DNA: two alleles are visible at 264 and 276 base pairs. Semiquantitative PCR showed that the 264-base pair allele was located on the idic(X)(q13), visible as a peak height ratio of 1.86. (d) After digestion, only the 276-base pair peak was present, showing that the isodicentric chromosome was active.

The number of CAG tandem repeats allowed differentiating the allele corresponding to the idic(X)(q13) from the allele pertaining to the normal X chromosome. Initially, we performed a semiquantitative PCR analysis of the genomic DNA on a capillary electrophoresis system, obtaining a fluorescence signal for each allele on the locus for the *HUMARA *gene, shown as a peak and area under the curve. Since the amplified region was present in two copies on the isodicentric X chromosome, the assay showed a higher peak with a ratio close to 2 as compared to the height of the two separate alleles (Figure 2(c)). The XCI patterns revealed that the idic(X)(q13) belonged to the 100% active X chromosome (Figure 2(d)). The genomic DNA from a healthy volunteer served as a control to validate the experiment (Figures 2(a) and 2(b)).

## 3. Discussion

The idic(X)(q13) is a rare cytogenetic abnormality generally associated with ring sideroblasts in neoplastic myeloid pathologies [6, 7]. Several authors [4, 6, 8] suggested that the *ABCB7* gene, located on the deleted Xq13 region in idic(X) positive cases and involved in the transportation of iron from mitochondria to cytosol in the erythroblasts, is lost exclusively on the active X chromosome. Moreover, a rare inherited X-linked sideroblastic anemia with spinocerebellar ataxia, characterized by anaemia with mitochondrial iron accumulation in the bone marrow erythroblasts, is caused by mutations in *ABCB7* gene. These defects of *ABCB7* would be the underlying cause of the common ring sideroblasts in these patients.

It is also feasible that the consequence of the presence of an idic(X)(q13) would be different if it occurs in the active or inactive X chromosome. More than 30 cases of idic(X)(q13) have been reported. In 14 cases an analysis of X inactivation was performed [4, 6, 9, 10], but only in two cases there are data available regarding the presence of ring sideroblasts. In addition, seven cases where the idic(X)(q13) was the inactive X chromosome were considered as myeloproliferative neoplasias [4, 9] without ring sideroblasts.

In summary, in patients with myeloid malignancies and idic(X)(q13), an analysis of methylation status of X chromosome would be recommended for determining any preferential inactivation. Further studies are needed to assess whether the activation state of idic(X)(q13) is correlated with the presence of ring sideroblasts and, as such, with the underlying pathology.
